# Virtual Communities of Practice: Overcoming Barriers of Time and Technology

**DOI:** 10.2196/jmir.3400

**Published:** 2014-07-29

**Authors:** Kieran Walsh, Stephen Barnett

**Affiliations:** ^1^BMJ LearningLondonUnited Kingdom; ^2^General Practice Academic UnitSchool of Science Medicine and HealthUniversity of WollongongWollongongAustralia

**Keywords:** medical education, community of practice, time, technology

## Letter

Barnett et al should be congratulated for their fascinating report into the use of virtual communities of practice for family physician training [1]. It is interesting that the respondents reported time as a barrier to their participation in the community of practice. Undoubtedly, it would have taken them time to take part, but the amount of time needed should be placed in context and compared with the time needed to partake in face-to-face communities of practice. Face-to-face participation would take far more time—not least the time required to travel to the venue of the community of practice meeting. So in truth, virtual communities of practice are far more likely to be time saving. However the feeling that time was a barrier cannot be dismissed out of hand. The fact that trainees felt it to be a barrier may say more about how the medical education community views the virtual world. Do we take learning in virtual communities as seriously as we take face-to-face learning? What would be the response of a tutor or trainer if a trainee requested study leave to participate in online education? Would they be as accommodating as they would be to a trainee who requested leave to attend an event? Is virtual learning something that trainers and trainees see as an activity to be undertaken in users’ own time? These questions are important—not least because time is money in all areas of life and not least in medical education. [2]

Second, there was the issue of technology and some users feeling less comfortable using chats and wondering whether webinars might be made available in future generations of the virtual community. With the increasing availability of broadband Internet and the players required for creating and watching webinars, there is no reason why this cannot happen. The users may also discover unexpected benefits from webinars. Foremost among these might be the ability to learn communication and team-working skills, which they will be able to do much more easily by means of watching and interacting with videos. This might be particularly useful for users who have not trained in Australia.

*Kieran Walsh*

## Author’s Response

Dr Walsh makes two very good points in his letter; firstly, that the perception of online time needs to be offset against the time taken for face-to-face training and secondly, that webinars are a viable option for online training.

Regarding the perception of time, in a training context this fits well with the findings from the ConnectGPR study that it is important that the training is recognized, rather than just being an “extra”. If the training time is recognized so that it offsets face-to-face time, then perhaps that perceived time barrier will diminish.

In terms of webinars, in fact the ConnectGPR project is continuing in Australia, and for the last 12 months we have been running regular webinars, rather than webchats, which have been very well received. This year, our project is to try to supply enough webinar training that is live and then available later as a recording, that registrars will be able to offset face-to-face workshop time by participating in these online events. Whilst face-to-face workshops are important and will never be completely replaced, for a training program that covers 160,000 square kilometres, there are potentially significant savings to be made in workshop attendance if even some of the face-to-face time can be replaced with online training.

*Stephen Barnett*
